# Novel Trajectories of Bromocriptine Antidiabetic Action: Leptin-IL-6/ JAK2/p-STAT3/SOCS3, p-IR/p-AKT/GLUT4, PPAR-γ/Adiponectin, Nrf2/PARP-1, and GLP-1

**DOI:** 10.3389/fphar.2018.00771

**Published:** 2018-07-18

**Authors:** Enji Reda, Sherifa Hassaneen, Hanan S. El-Abhar

**Affiliations:** ^1^Department of Pharmacology, National Organization for Drug Control and Research, Cairo, Egypt; ^2^Department of Pharmacology and Toxicology, Faculty of Pharmacy, Cairo University, Cairo, Egypt

**Keywords:** bromocriptine, sitagliptin, insulin signaling, IL-6, leptin, JAK2/STAT3, PPAR-γ, Nrf2

## Abstract

Bromocriptine (BC), a sympatholytic dopaminergic D2 receptor agonist, has been comprehensively used in clinic to treat Parkinson’s disease (PD) and prolactinomas. Besides, BC represents a novel therapeutic option in type 2 diabetes (T2DM); however, the precise mechanisms are not completely unveiled. Hence, the objective of the current work is to clarify the potential molecular pathways of the insulin sensitizing effect of BC in the skeletal muscle of diabetic rats and to evaluate its possible interaction with sitagliptin (SG) as an add-on therapy. Here experimental model impersonates unhealthy dietary habit and T2DM was adopted, in which rats were fed high caloric diet of fat and fructose for 6 weeks followed by a single sub-diabetogenic dose of streptozotocin (STZ) (35 mg/kg; HF/Fr/STZ). Diabetic rats were treated with BC, SG at two dose levels (SG10 and SG20) and combination of BC + SG10 for 2 weeks. BC successfully corrected glucose/lipid profile, as well as leptin and GLP-1. On the muscular molecular level, BC curtailed the inflammatory signal IL-6/JAK2/*p*-STAT3/SOCS3, while enhanced the PPAR-γ/adiponectin signaling, resulting in activation of the insulin signaling pathway (*p*-IR/*p*-AKT/GLUT4). Moreover, BC confirmed its antioxidant capabilities by altering Nrf2 and PARP-1; the study also highlighted novel mechanisms for SG as well. On almost all tested parameters/pathways, the combination regimen surpassed each drug alone to reach a comparable level to the high dose of SG. In conclusion, our finding shed some light on novel anti-diabetic mechanisms of BC. The study also points to the potential use of BC as an adds-on to standard anti-diabetic therapies.

## Introduction

Over the past century, technological advances and improved economic status have shifted the energy balance toward a more sedentary lifestyle with increased consumption of westernized diet (Johnson et al., 2009; Al-Goblan et al., 2014). Such fat rich diet along with fructose in soft drinks has likely contributed to the dramatic up surge in the prevalence of overweight and obesity (Park et al., 2007) along with the increased risk of developing I/R and T2DM (Kahn et al., 2006).

Following a meal, approximately one third of ingested glucose is taken up by the liver and the rest by peripheral tissues, primarily skeletal muscle, via an insulin dependent mechanism. However, in I/R status, insulin-stimulated glucose disposal in skeletal muscle is markedly blunted because of the impaired insulin signaling and multiple post-receptor intracellular defects (DeFronzo, 1997; Bouzakri et al., 2005).

Ample evidence stated that patients with T2DM have an increased risk of developing PD and share similar dysregulated pathways, suggesting common underlying pathological mechanisms (Lima et al., 2014; Santiago and Potashkin, 2014; Athauda and Foltynie, 2016). Dopamine is an eminent regulator of food intake, energy expenditure, and goal-oriented behaviors. Hence, reduced dopamine signal transduction causes an overeating together with decreased energy expenditure ending up with obesity (Davis et al., 2009; Thanos et al., 2011).

Bromocriptine, a sympatholytic D2 receptor agonist, is an old drug used clinically for over 30 years to treat patients with PD and prolactinomas. Nevertheless, the new quick-release formulation of BC (bromocriptine-QR; Cycloset) was approved by the FDA in May 2009, as a novel therapeutic option in T2DM (Via et al., 2010). BC is unique in that it does not have a specific receptor to mediate its action on glucose and lipid metabolism; rather, it acts by resetting dopaminergic and sympathetic tone within the central nervous system (CNS). It is believed that morning daily administration of BC augments low hypothalamic dopamine (DA) levels to act on D2 receptor and inhibits excessive sympathetic tone within the CNS. These effects reduce post-prandial plasma glucose levels by suppressing hepatic glucose production and gluconeogenesis (DiCostanzo et al., 2006; Defronzo, 2011; Ezrokhi et al., 2012). Furthermore, BC lower the plasma insulin level (Cincotta et al., 1991). Numerous studies have demonstrated that BC improves lipid profile, produces significant reductions in body fat mass, reduces TGs, and FFAs, and convalesces glucose homeostasis (Meier et al., 1992; Pijl et al., 2000; Kok et al., 2006). Reduction of glucose and insulin levels as well as impaired lipids are the proposed mechanism of action of BC to improve glucose tolerance (Shivaprasad and Kalra, 2011; Lamos et al., 2016; Derkach et al., 2017). However, the molecular mechanisms by which BC mediate its effect on T2DM in the skeletal muscle is not completely unveiled.

Sitagliptin (SG), the first DPP4 inhibitor to be approved in the United States for treatment of T2DM, is used as a monotherapy or in combination with other drugs. It inhibits the enzymatic degradation and inactivation of the incretins, GLP-1 and GIP. These incretins augment glucose-induced insulin secretion after meals.

Glucagon-like peptide-1 is mainly secreted from intestinal L cells and has beneficial effects on glucose homeostasis. It suppresses glucagon release, delays gastric emptying, increases satiety, and stimulates β-cell regeneration (Drucker, 2006; Gallwitz, 2007).

Furthermore, GLP-1 is also produced in the brain (Kappe et al., 2012) and has neuroprotective, neurogenic action on different brain regions (Parthsarathy and Hölscher, 2013). Besides, GLP-1 has anti-inflammatory action that were evidenced on different diseases associated with chronic inflammation either in peripheral or central tissues (Lee and Jun, 2016). Recently, there are several inquiries on neuroprotective effect of DPP-IV inhibitors as a novel anti-parkinsonian approach, which mediated by their neuroprotective, neuroregenerative properties besides their antioxidant anti-inflammatory and anti-apoptotic signaling pathways (Badawi et al., 2017).

To this end, the aim of our study was to unveil some of the potential anti-diabetic mechanisms of BC and its possible use as an add on therapy to anti-diabetic drugs using a type 2 diabetic model.

## Materials and Methods

### Animals

Adult male Wistar rats (80–120 g; aged 4–6 weeks) were obtained from the Animal House Colony of the National Organization for Drug Control and Research (NODCAR; Cairo, Egypt). They were housed in polypropylene cages (5 rats/cage) with wire covers and hardwood chips bedding, under specific pathogen-free conditions in facilities maintained at controlled environmental conditions (21–24°C and 40–60% humidity), and equal light-dark cycles (12/12 h light/dark cycle). The bedding was changed daily in the morning after moving the rats to a new clean cage to avoid the offensive odor. Rats had free access to standard rodent chow was designed at National Research Institute for Nutrition (Giza, Egypt), according to National Nutrition Database for Standard Reference. The diet ingredients were purchased from El-Nasr Pharmaceutical Chem. (New Maadi, Cairo, Egypt) and it consists of fat (5%), carbohydrate (60%), protein (21%), fibers (3%), and vitamins and minerals (1%). The animals were allowed free access to tap drinking water for 1 week prior to dietary manipulation.

### Ethics Statement

Experimental design and animal handling were carried out according to the Guide for the Care and Use of Laboratory Animals (NIH, 1996) and after the approval of the Ethics Committee of Faculty of Pharmacy, Cairo University (Cairo, Egypt; PT:PO.34.6).

### Drugs and Chemicals

Bromocriptine mesylate (BC) was a gift from Amoun Co. (Cairo, Egypt), whereas SG phosphate was obtained from Merck Sharp & Dohme Ltd. (Pavia, Italy) as the Januvia^®^ tablet. STZ was purchased from Sigma-Aldrich Co. (St. Louis, MO, United States), while long-acting insulin (Mixtard) was obtained from Novo Nordisk Co. (Copenhagen, Denmark). The diet ingredients, such as cholesterol and fructose were procured from El-Nasr Chemical Co. (Cairo, Egypt) and Universal International Pharmaceutical Co. (Cairo, Egypt), respectively. Lard was obtained from commercial sources and other chemicals used were of analytical grades. BC was freshly prepared in DMSO (3%), whereas SG was prepared in distilled water. Both drugs were administered in the morning.

### Induction of Type 2 Diabetes

The model was induced according to a previous study by Schaalan et al. (2009). Rats were divided into two main groups; the first group (*n* = 10) served as the normal control group and rats were fed standard rodent chow and tap drinking water *ad libitum*.

In the second group (*n* = 80), rats were fed 10% of their body weight with HFD, which consists of fat (20% saturated animal fat and 1% cholesterol powder), carbohydrate (60%), protein (21%), fibers (3%), and vitamins and minerals (1%) (Reed et al., 2000). These animals had free access to drinking water containing 20% (w/v) fructose. The diet regimen continued for a period of 6 weeks; during the sixth week, the second group was injected a daily single dose of long-acting human insulin (Mixtard) (0.5 IU/kg, i.p) to augment a state of I/R; while the 1st group was given normal saline. On the seventh week, rats received a single i.p injection of a sub-diabetogenic dose of freshly prepared STZ (35 mg/kg) in a citrate buffer (0.09M, pH 4.8) after an over-night fasting (Reaven and Ho, 1991), while the respective control rats were given the vehicle only (citrate buffer, pH 4.5). To overcome STZ-induced hypoglycemia during the first 24 h, rats were given 5% glucose solution to drink instead of tap water. The feeding regimen was stopped after STZ injection and 1 week later animals that fulfill the required criteria (persistent blood glucose levels between 200 and 350 mg/dl, hyperinsulinemia, insulin resistant, and dyslipidemia), were used in the study and served as diabetic rats (HF/Fr/STZ) (**Scheme 1**).

**SCHEME:**
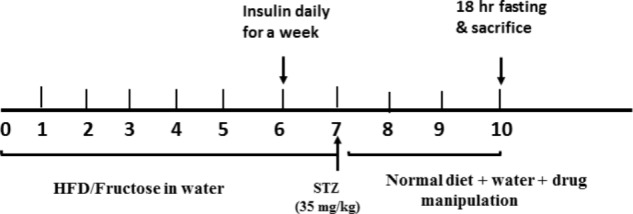
Experimental design timeline.

### Determination of Intraperitoneal Glucose Tolerance Test (IPGTT)

One week after STZ administration, normal and diabetic rats were fasted for 6 h before the test. Droplets of blood were collected from the tail vein at the 0 time to determine the fasting glucose level, and then animals were injected with glucose solution (2 g/kg glucose powder was dissolved in distilled water, i.p). Blood samples were withdrawn at 30, 60, 90, and 120 min to evaluate the glucose concentration using glucose measuring device (Accu-check Compact) (Schaalan et al., 2009). I/R was reflected by the IPGTT and was further confirmed by the fasting hyperinsulinemia and the HOMA-I/R value.

### Experimental Design

Rats were allocated into six groups; the first group represents the non-diabetic normal control groups (NC) that were fed with the standard rodent chow, whereas the diabetic rats (HF/Fr/STZ) were further subdivided into five subgroups. Group 2 includes the untreated diabetic rats (diabetic control, DC), group 3 were rats treated with BC (20 mg/kg/i.p, BC) (Thanos et al., 2011). In groups 4, 5, and 6 rats were treated with SG (10 mg/kg; orally, SG_10_), SG (20 mg/kg/orally, SG_20_) (Nade et al., 2015; Badawi et al., 2017), and a combination of BC + SG_10_, respectively. Animals in groups 1 and 2 received the vehicle (3% DMSO, i.p) and all animals were allowed free access to standard chow and water *ad libitum*. All treatments were administered for 2 weeks and the last dose of any treatment was given 24 h before the time of carnage. Animals were fasted 18 h before time of death to minimize the feeding-induced variations in lipid and glucose pattern.

### Collection of Serum Samples for Analysis

At the end of experiment, blood was collected from the tail vein and centrifuged (4,000 rpm, 4°C, 20 min) to separate sera that were used to determine glucose, TGs, TC and HDL-C using Randox colorimetric reagent kits (Antrim, United Kingdom). LDL-C was calculated according to the Friedewald equation (Friedewald et al., 1972):

$$\begin{array}{c} LDL-C\;(mg/dl)\;=\;TC\;-\;[HDL-C+ (5)] \end{array}$$

The serum levels of insulin (RayBiotech, Inc., Norcross, GA, United States; Cat #: ELR-Insulin), fructosamine (MyBioSource, Inc., San Diego, CA, United States; Cat #: MBS2601586), leptin (RayBiotech, Inc., Norcross, GA, United States; Cat #: ELR-Leptin), adiponectin (Invitrogen, Carlsbad, CA, United States; Cat #: KRP0041), and GLP-1 (LifeSpan BioSciences, Inc., Seattle, WA, United States; Cat #: LS-F23155) were analyzed using commercially available ELISA kits according to the manufacturers’ instructions.

The HOMA-I/R was determined using the following formula (Matthews et al., 1985):

HOMA-I/R = (serum glucose (mmol/L) × serum insulin (μIU/ml))/22.5

### Tissue Extracts for Analysis

After blood collection, animals were euthanized using deep sodium pentobarbital anesthesia and soleus skeletal muscle was dissected out, washed thoroughly with ice-cold PBS (0.02 mol/l, pH 7.0–7.2) to remove excess blood. Muscle tissue (0.5 g) was homogenized in 5 ml of PBS and the resulting suspension was centrifuged (4,000 rpm, 4°C, 20 min). The supernatant was collected, divided into aliquots, and preserved at -80°C until estimation of the following biomarkers.

#### Assessment of Soleus Skeletal Muscle Contents of IL-6/JAK2*/p*-STAT3/SOCS3

An aliquot was exposed to two repeated freeze-thaw cycles to further break the cell membranes, then centrifuged at 5,000 × *g* for 5 min and stored at -20°C. The corresponding ELISA rat kits were used to assess the skeletal contents of IL-6 (RayBiotech, Inc., Norcross, GA, United States; Cat #: ELR-IL6), JAK2 (MyBioSource, Inc., San Diego, CA, United States; Cat #: MBS7221943), STAT3[pY705] (Invitrogen, Carlsbad, CA, United States; Cat #: KHO0481), and SOCS3 (Cloud-clone-Corp, Katy, TX, United States; Cat #:SEB684Ra).

#### Assessment of Soleus Skeletal Muscle Insulin Signaling Pathway

In another aliquot, ELISA rat kits were used for the estimation of IR [pYpY1162/1163] (Invitrogen, Carlsbad, CA, United States; Cat #: KHR9131) and AKT [pS473] (MyBioSource, Inc., San Diego, CA, United States; Cat #: MBS7221943), as well as GLUT4 (Elabscience, Houston, TX, United States; Cat #: E-EL-R0430).

#### Assessment of Soleus Skeletal Muscle Contents of Nuclear Factor [Erythroid-Derived 2]-Like 2 (Nrf2), PARP-1, and PPAR-γ

The above parameters were assayed according to the manufacturer instructions of the related ELISA kit; Nrf2 (MyBioSource, Inc., San Diego, CA, United States; Cat #: MBS752046), PARP-1 (LifeSpan BioSciences, Inc., Seattle, WA, United States; Cat #: LS-F27548), and PPAR-γ (LifeSpan BioSciences, Inc., Seattle, WA, United States; Cat #: LS-F4266).

For all parameters assessed using the ELISA technique the following was carried out; standards, control, and samples were by pipetted into the corresponding microplate wells. The plate was covered and incubated; the amount of sample, time of incubation, and temperature was carried out according to the manufacturer instructions. Wells were then washed with wash buffer solution, and the wells were tapped, then any remaining liquid is removed by aspiration. This was followed by the addition of the detection antibody solution (secondary antibody or detection reagent A) to each well, and then the plate was covered and incubated. After the removal of excess secondary antibody, detection reagent B is added to each well to complete the four member sandwich. After incubation, stabilized chromogen or tetramethylbenzidine (TMB) substrate solution was added, this would interact with the bound enzyme to produce a blue color. The stop solution was finally added to each plate, with the conversion of the blue color into yellow. The absorbance was read at 450 nm, using ELISA reader (Biomak, Beckman, United States).

### Statistical Analysis

Data were expressed as mean ± SD of six animals. Statistical comparisons between means were carried out using one-way analysis of variance (ANOVA), followed by Student-Newman–Keuls test. The statistical significance of difference was considered at *P* < 0.05. The GraphPad Prism v5.0 (GraphPad Prism Inc., La Jolla, CA, United States) was used to analyze and present all the data.

## Results

### Intraperitoneal Glucose Tolerance Test (IPGTT)

**FIGURE 1:**
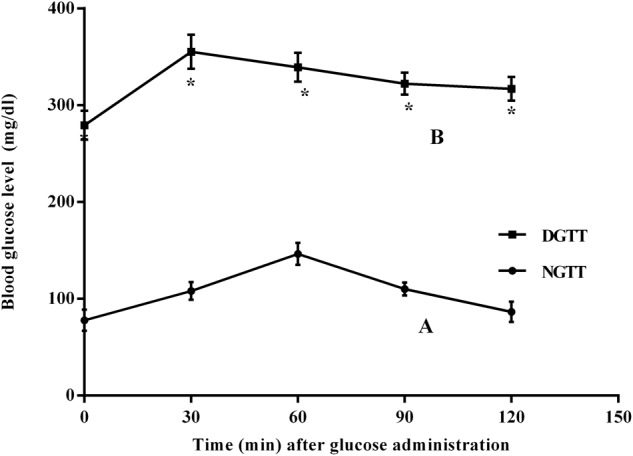
Intraperitoneal glucose tolerance test (IPGTT) curves in normal (NGTT) and diabetic (DGTT) rats. Blood samples at 0, 30, 60, 90, and 120 min of **(A)** normal and **(B)** diabetic rats after receiving glucose solution (2 g/kg; i.p). Values are means of 6 rats ± SD; as compared with normal control (NC; ^∗^) using unpaired Student’s *t*-test at *P* < 0.05.

Glucose intolerance/insulin resistance (I/R) was shown in **Figure 1**, where the fasting blood glucose level of the diabetic rats increased by 3.6-fold, as compared to normal group. This level peaked 30 min post-glucose administration, and then started to decline gradually, yet it was markedly higher than that of normal rats, confirming, thus, a state of glucose intolerance/insulin resistance (I/R).

### Glucose Homeostasis-Related Parameters

**FIGURE 2:**
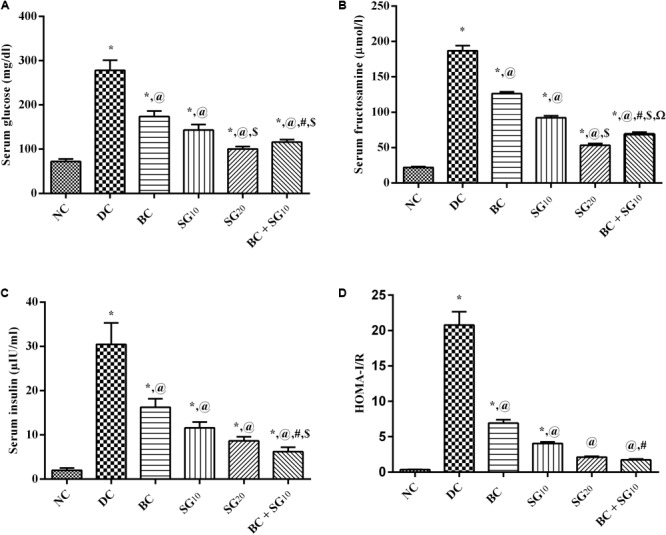
Effect of BC and/or SG on glucose homeostasis. Effect of 2 weeks daily administration BC (20 mg/kg, i.p), SG_10_, SG_20_ (10 and 20 mg/kg, p.o) and their combination (BC + SG_10_) on serum **(A)** glucose, **(B)** fructosamine, **(C)** insulin, and **(D)** HOMA-I/R in HF/Fr/STZ diabetic rats. Values are means of 6–10 rats ± SD. As compared with normal control (NC; ^∗^), diabetic control (DC; @), BC (#), SG_10_ ($), and SG_20_ (Ω) treated groups (one-way ANOVA followed by Newman–Keuls multiple comparison test) at *P* < 0.05. BC, bromocriptine; SG, sitagliptin.

The diabetic model elevated significantly the fasting serum glucose, fructosamine, insulin, and HOMA-I/R by 3.9-, 8.5-, 15-, and 64-folds, respectively, compared to the normal control rats (**Figures 2A–D**). All treatment regimens leveled off the assessed parameters to different levels, as compared to the diabetic rats. Addition of SG_10_ to BC caused a further reduction in all parameters to a level that was significant from each drug alone and was almost comparable to that of SG_20_.

### Lipid Profile-Related Parameters

**FIGURE 3:**
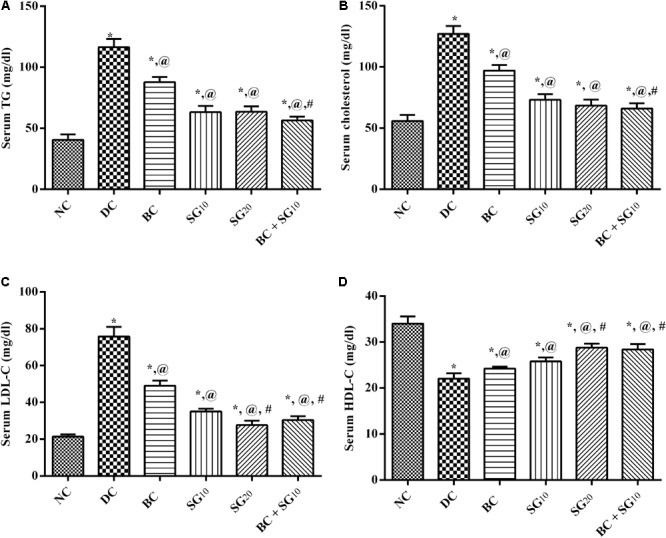
Effect of BC and/or SG on serum lipid profile. Effect of 2 weeks daily administration BC (20 mg/kg, i.p), SG_10_, SG_20_ (10 and 20 mg/kg, p.o) and their combination (BC + SG_10_) on serum **(A)** TGs, **(B)** TC, **(C)** LDL-C, and **(D)** HDL-C in HF/Fr/STZ diabetic rats. Values are means of 6–10 rats ± SD. As compared with normal control (NC; ^∗^), diabetic control (DC; @), BC (#), SG_10_ ($), and SG_20_ (Ω) treated groups (one-way ANOVA followed by Newman–Keuls multiple comparison test) at *P* < 0.05. BC, bromocriptine; SG, sitagliptin; TGs, triglycerides; TC, total cholesterol; LDL-C, low-density lipoprotein; HDL-C, high-density lipoprotein.

As depicted in **Figure 3**, the HF/Fr/STZ model markedly elevated the serum levels of (A) TGs, (B) TC, and (C) LDL-C, but abated that of (D) HDL-C, as compared to normal group. Nevertheless, all treatments improved significantly the altered lipid panels with the combined regimen showing the best effect compared to diabetic untreated group.

### Changes in Serum/Muscular Leptin-IL-6/JAK2/*p*-STAT3/SOCS3

**FIGURE 4:**
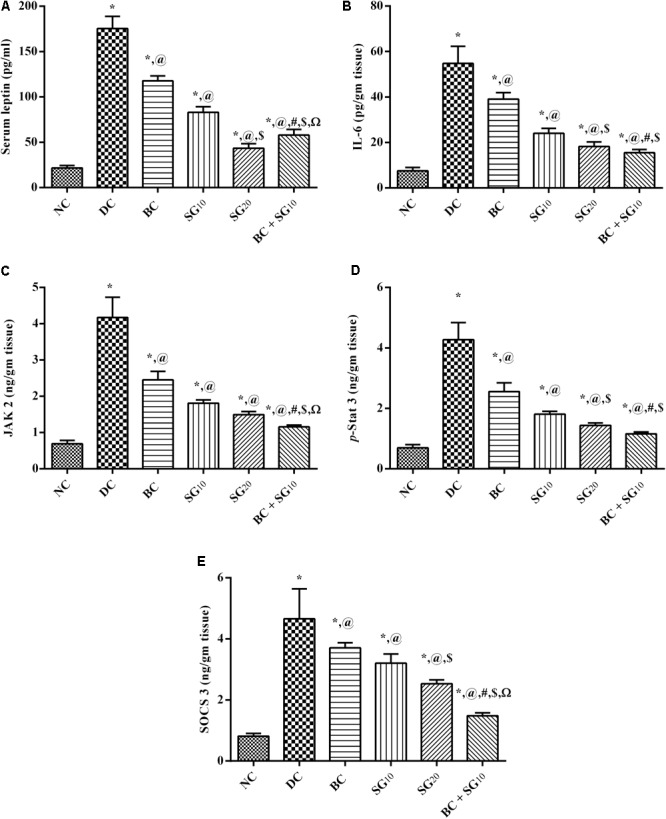
Effect of BC and/or SG on Leptin-IL-6/JAK2/*p*-STAT3/SOCS3 signaling in the skeletal muscle. Effect of 2 weeks daily administration BC (20 mg/kg, i.p), SG_10_, SG_20_ (10 and 20 mg/kg, p.o) and their combination (BC + SG_10_) on **(A)** serum leptin, as well as muscular contents of **(B)** IL-6, **(C)** JAK2, **(D)** *p*-STAT3, and **(E)** SOCS3 in HF/Fr/STZ diabetic rats. Values are means of 6–10 rats ± SD. As compared with normal control (NC; ^∗^), diabetic control (DC; @), BC (#), SG_10_ ($), and SG_20_ (Ω) treated groups (one-way ANOVA followed by Newman–Keuls multiple comparison test) at *P* < 0.05. BC, bromocriptine; SG, sitagliptin*;* IL-6, interleukin 6; JAK2, Tyrosine protein kinase Janus kinase 2; STAT3[pY705], *p*-STAT3, phosphorylated signal transducer and activator of transcription 3 at tyrosine residue 705; SOCS3, suppressor of cytokine signaling 3.

As presented in **Figure 4**, the diabetic model boosted serum (A) leptin and the (B) muscular inflammatory cytokine IL-6, compared with the normal control values. As a consequence (C) JAK2 and its downstream (D) *p*-STAT3 were increased, as well as the (E) compensatory molecule SOCS3, compared to normal group. On the other hand, BC displayed a significant reduction in IL-6 and leptin, as well as the JAK2/*p*-STAT3/SOCS pathway. The same pattern was mediated by the administration of SG_10_ and SG_20_, with the high dose showing better lowering effect. Moreover, diabetic animals treated with combined regimen showed a noticeable decline in IL-6/leptin/JAK2/*p*-STAT3/SOCS3, compared to single drugs and diabetic untreated group.

### Changes in Muscular Insulin Signaling Pathway (*p*-IR/*p*-AKT/GLUT4)

**FIGURE 5:**
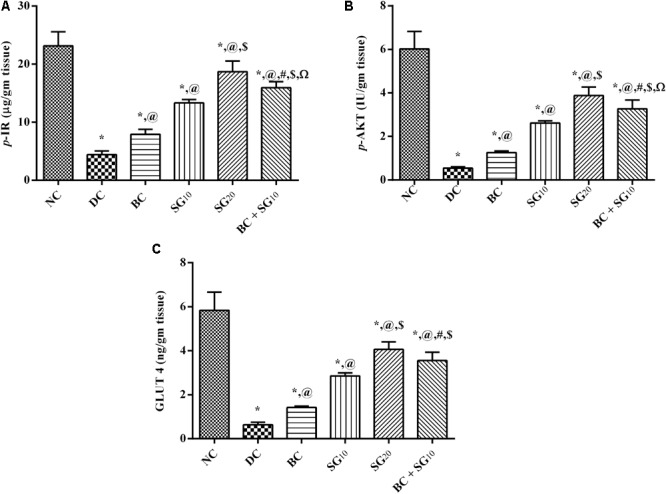
Effect of BC and/or SG on insulin signaling pathway (*p*-IR/*p*-AKT/GLUT4) in the skeletal muscle. Effect of 2 weeks daily administration BC (20 mg/kg, i.p), SG_10_, SG_20_ (10 and 20 mg/kg, p.o) and their combination (BC + SG_10_) on soleus skeletal muscle content of **(A)** *p*-IR, **(B)** *p*-AKT, and **(C)** GLUT4 in HF/Fr/STZ diabetic rats. Values are means of 6–10 rats ± SD. As compared with normal control (NC; ^∗^), diabetic control (DC; @), BC (#), SG_10_ ($), and SG_20_ (Ω) treated groups (one-way ANOVA followed by Newman–Keuls multiple comparison test) at *P* < 0.05. BC, bromocriptine; SG, sitagliptin; *p*-IR, phosphorylated insulin receptor [pYpY1162/1163]; *p*-AKT, phosphorylated protein kinase B [pS473]; GLUT4, glucose transporter 4.

The model, as depicted in **Figure 5**, caused a tremendous decrease in the (A) muscular *p*-IR and (B) *p*-AKT leading to a decrease in (C) GLUT4, compared with normal control. Meanwhile, these effects were reversed almost in all treated animals in the order of BC, SG_10_, and BC/SG_10_, the latter was comparable to the effect of SG_20_.

### Changes in Muscular PPAR-γ and Serum Adiponectin

**FIGURE 6:**
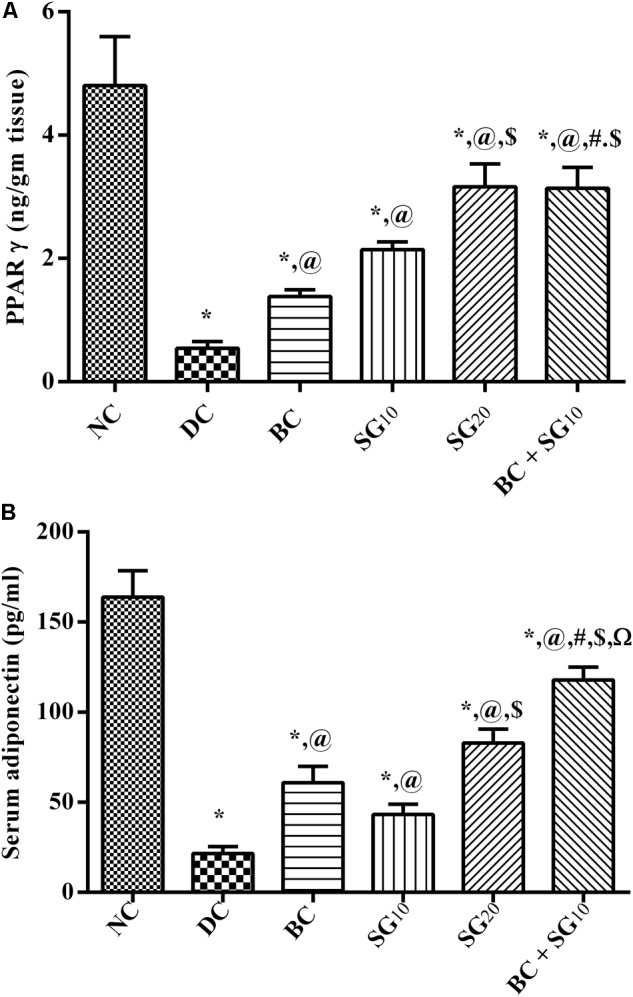
Effect of BC and/or SG on PPAR-γ/Adiponectin signaling in the skeletal muscle. Effect of 2 weeks daily administration BC (20 mg/kg, i.p), SG_10_, SG_20_ (10 and 20 mg/kg, p.o) and their combination (BC+SG_10_) on soleus skeletal muscle content of **(A)** PPAR-γ and **(B)** serum adiponectin in HF/Fr/STZ diabetic rats. Values are means of 6–10 rats ± SD. As compared with normal control (NC; ^∗^), diabetic control (DC; @), BC (#), SG_10_ ($), and SG_20_ (Ω) treated groups (one-way ANOVA followed by Newman–Keuls multiple comparison test) at *P* < 0.05. BC, bromocriptine; SG, sitagliptin; PPAR-γ, peroxisome proliferators-activated receptor gamma.

The content of PPAR-γ in soleus skeletal muscle was notably decreased (88.5%) in the diabetic model relative to the normal control; this inhibition was linked to the reduction in serum adiponectin (86.8%) (**Figures 6A,B**). Both BC and SG in the 2 dose levels intensified both PPAR-γ content and serum level of adiponectin in an ascending order. Moreover, combined regimen produced a profound improvement in the two parameters, as compared to either drug alone and to the diabetic control.

### Changes in Muscular Nrf2 and PARP-1 Signaling in Skeletal Muscle

**FIGURE 7:**
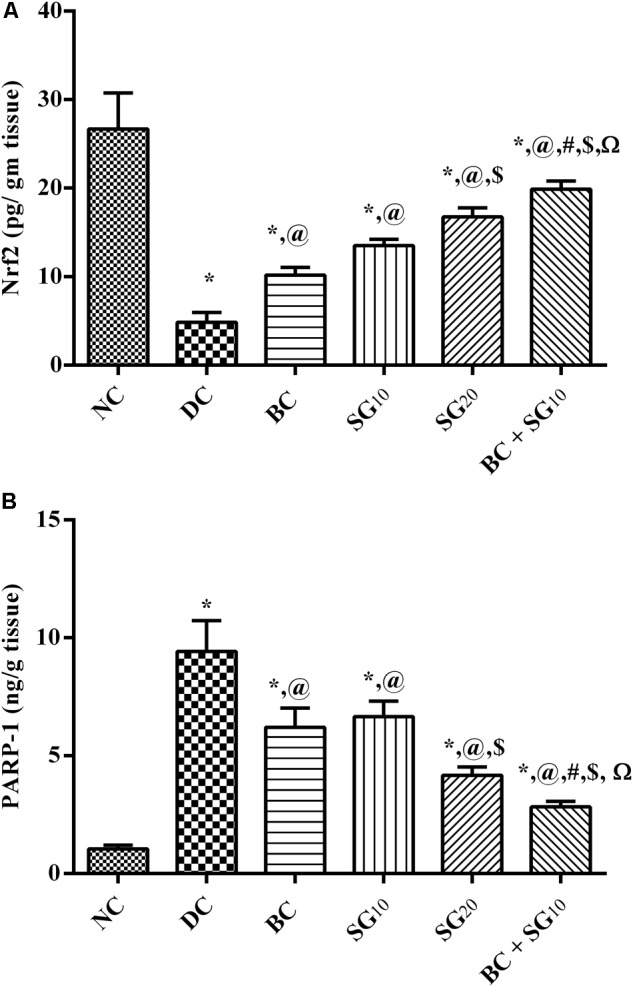
Effect of BC and/or SG on Nrf2/PARP-1 signaling in the skeletal muscle. Effect of 2 weeks daily administration BC (20 mg/kg, i.p), SG_10_, SG_20_ (10 and 20 mg/kg, p.o) and their combination (BC + SG_10_) on soleus skeletal muscle content of **(A)** Nrf2 and **(B)** PARP-1 in HF/Fr/STZ diabetic rats. Values are means of 6–10 rats ± SD. As compared with normal control (NC; ^∗^), diabetic control (DC; @), BC (#), SG_10_ ($), and SG_20_ (Ω) treated groups (one-way ANOVA followed by Newman–Keuls multiple comparison test) at *P* < 0.05. BC, bromocriptine; SG, sitagliptin; Nrf2, Nuclear factor [erythroid-derived 2]-like 2; PARP-1, poly [ADP-ribose] polymerase 1.

As a consequence to the inhibited *p*-AKT, the diabetic insult abated the transcriptional factor Nrf2, while it caused ninefold increase in PARP-1 (**Figures 7A,B**). In contrast, BC treatment was able to partially revert the diabetic effect. Similarly, the 2 doses of SG opposed the diabetic alterations dose dependently. In addition, combined treatment with BC and SG_10_ succeeded to produce the best effect among the treated groups.

### Changes in Serum GLP-1

**FIGURE 8:**
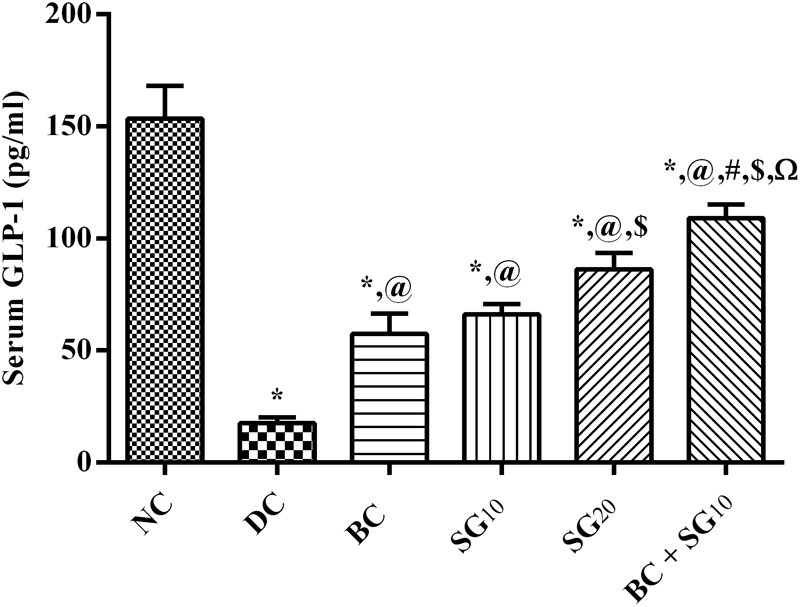
Effect of BC and/or SG on serum GLP-1. Effect of 2 weeks daily administration BC (20 mg/kg, i.p), SG_10_, SG20 (10 and 20 mg/kg, p.o) and their combination (BC + SG_10_) on serum GLP-1 in HF/Fr/STZ diabetic rats. Values are means of 6–10 rats ± SD. As compared with normal control (NC; ^∗^), diabetic control (DC; @), BC (#), SG_10_ ($), and SG_20_ (Ω) treated groups (one-way ANOVA followed by Newman–Keuls multiple comparison test) at *P* < 0.05. BC, bromocriptine; SG, sitagliptin; GLP-1, glucagon-like peptide-1.

In the diabetic untreated animals, the incretin hormone level was depleted to reach 11.4% only relative to the normal control value, as shown in **Figure 8**. However, BC elevated GLP-1 (3.3-fold), as well as SG_10_ (3.8-fold), and SG_20_ (4.9-fold), in relation to the insult. Once again, the greatest elevation effect was observed in the combination treated group to be 6.2-fold the diabetic value.

## Discussion

The present study was first conducted to clarify the potential molecular pathways of the insulin sensitizing effect of BC in the skeletal muscle of diabetic rats. Moreover, it illustrated that SG can act via other mechanisms than its DPP4 inhibitory effect to improve I/R and diabetes. Besides, the study highlighted the beneficial effect of BC when used as an adds-on therapy with the low tested dose of SG.

The daily morning administration of BC improved glucose/insulin homeostasis, as well as lipid profile to support the findings of previous studies (Cincotta et al., 1999; Ezrokhi et al., 2012; Derkach et al., 2017). However, the effect of BC on skeletal muscle, a core peripheral organ in I/R/diabetes, has not been exploited before.

The current model was verified previously by our laboratory and by others reporting that chronic consumption of high fat and fructose induce hyperglycemia, hyperinsulinemia, and dyslipidemia leading to the development of I/R state. These alterations are induced by several mechanisms including chronic inflammation, adipocytokines, and oxidative stress (Wei et al., 2008; Lozano et al., 2016). Skeletal muscles, *per se*, have a critical role in the production and secretion of several cytokines or myokines that exert autocrine, paracrine, or endocrine effects (Pedersen et al., 2007; Lightfoot and Cooper, 2016). On the molecular level, BC opposed the diabetic effect and turned off the JAK2/*p*-STAT3/SOCS3 axis by abating its two triggers; viz., IL-6 and leptin (Alvarez-Aguilar et al., 2013). These cytokines stimulate this cascade upon binding to their muscular cytokine receptors (Lubis et al., 2008; Sarvas et al., 2013; Wunderlich et al., 2013), to phosphorylate JAK2 and STAT3. As a consequence, their negative feedback regulator SOCS3 was also increased as reported in our study and previous ones (Wei et al., 2008; Mashili et al., 2013; Kim et al., 2017). However, controversial explanations were offered, where some studies referred this elevation to the ability of SOCS3 to counterbalance the inflammatory events (Jorgensen et al., 2013; Kim et al., 2017). Besides, increased SOCS3 was reported to inhibit leptin signaling, which in turn down regulates the leptin-regulated genes as those involved in fatty acid oxidation and mitochondrial function (Yang et al., 2012; Jorgensen et al., 2013). On the contrary, other studies found that SOCS3 also induces I/R by suppressing the phosphorylation of insulin receptor substrate 1 with the subsequent downstream signaling (Ueki et al., 2004; Sarvas et al., 2013). Hence, the ability of BC to reduce the JAK2/STAT3/SOCS3 axis can explain partially the improved insulin sensitivity and lipid profile reported herein. As a further documentation, BC increased the phosphorylated insulin receptor ([pYpY1162/1163]) with the subsequent phosphorylation of AKT and GLUT4 to signify the activated insulin signaling pathway.

Another tackled signaling pathway is the PPAR-γ/adiponectin pathway known to be silenced in diabetes (Hevener et al., 2003; Norris et al., 2003). This pathway was enhanced in the diabetic rats treated with BC to verify its anti-diabetic effect. Besides its beneficial role against diabetes, PPAR-γ also increases adiponectin (Combs et al., 2002; Iwaki et al., 2003), which orchestrates both glucose and lipid homeostasis (Tomas et al., 2002; Kadowaki et al., 2006); resulting in the reduction of lipid accumulation and improvement of insulin sensitivity. The role of PPAR-γ extended also to restitute DA, partially via protecting dopaminergic neurons (Randy and Guoying, 2007). These authors showed that pioglitazone in a PD model secured these neurons by its anti-inflammatory, and anti-oxidant properties.

Moreover, the third studied signaling pathway is the Nrf2/AKT cascade, which was reported earlier to be responsible for the cytoprotective and anti-oxidative effects of BC (Lim et al., 2008). In the present work, BC increased both Nrf2 and the *p*-AKT, which were both reduced by the current model. Apart from its insulin inter-relation (Uruno et al., 2013), *p*-AKT was reported to increase the transcriptional factor Nrf2 through the inactivation/phosphorylation of GSK-3 (Dieter, 2015). Decreased activity of Nrf2 is linked to the diabetes-associated oxidative stress, which in turn mitigates the antioxidant defense system to provoke I/R (de Haan, 2011; Tan et al., 2011). Moreover, the inhibition of Nrf2 activates PARP-1 to disturb glucose metabolism (Ganesh Yerra et al., 2013); Nrf2 positively regulates genes encoding enzymes involved in glycolysis and the pentose phosphate pathway (Dinkova-Kostova and Abramov, 2015). Additionally, over activation of PARP-1 inhibits glycolysis and causes mitochondrial impairment (Andrabi et al., 2014). Therefore, the BC-mediated insulin sensitivity is partly owed to the increased Nrf2 and the decreased PARP-1 to alleviate diabetes-induced oxidative stress and inflammation.

Another possible mechanism by which BC improved glucose/insulin homeostasis, as well as lipid profile is via increasing the GLP-1 level, which was decreased in diabetic rats. Besides its known mechanisms that regulate glucose homeostasis (Gallwitz, 2007; Lu et al., 2012), GLP-1 increases Nrf2 levels and its translocation into the nucleus resulting in improving the cellular antioxidant capacity of β-cells (Fernández-Millán et al., 2016). These findings support the present work as BC increased both GLP-1 and Nrf2.

The current study also highlighted the novel mechanisms for SG, the reference drug used, where apart from its known mechanism as a DPP4 inhibitor, SG abated the IL-6-leptin/JAK2/*p*-STAT3/SOCS3 signaling and Nrf2/PARP-1/AKT antioxidant pathway. These finding supports a previous study (Al-Rasheed et al., 2016), where SG reduced cardiac JAK2 and STAT3 phosphorylation in diabetic rats. This down-regulation could be attributed to the anti-inflammatory effect of SG by reducing the level of IL-6 (Al-Rasheed et al., 2016; Badawi et al., 2017).

Interestingly, the addition of BC with the low dose of SG showed a better effect on all tested parameters/pathways, when compared with each drug alone to reach a comparable level to the high dose of SG.

## Conclusion

In conclusion, we shed some light on novel mechanisms by which BC mediated its useful anti-diabetic effect. The study also points to the potential positive effect of BC as an adds-on therapy to the conventional antidiabetics. In addition, BC can be a useful medication for the treatment of diabetic patients with PD and vice versa. However, further studies are warranted to investigate other mechanisms of BC and its efficacy with other antidiabetics.

## Author Contributions

ER and HE-A conceived and designed the study. ER performed the experimental part and acquired and analyzed the data. SH drafted the article. HE-A led the research and revised and approved the article for submission.

## Conflict of Interest Statement

The authors declare that the research was conducted in the absence of any commercial or financial relationships that could be construed as a potential conflict of interest.

## Abbreviations

AKT [pS473]: *p*-AKT, phosphorylated protein kinase B at serine 473
BC: Bromocriptine
D2 receptor: Dopaminergic D2 receptor
DMSO: dimethyl sulfoxide
DPP4: dipeptidyl peptidase-4
FDA: Food and Drug Administration
FFAs: free fatty acids
GIP: glucose-dependent insulinotropic peptide
GLP-1: glucagon-like peptide-1
GLUT4: glucose transporter 4
GSK-3: glycogen synthase kinase 3
HDL-C: high-density lipoprotein
HF/Fr/STZ: High Fat/Fructose/STZ
HFD: high fat diet
HOMA-I/R: homeostasis model assessment index for insulin resistance
I/R: insulin resistance
IL-6: interleukin 6
IPGTT: intraperitoneal glucose tolerance test
IR [pYpY1162/1163]: *p*-IR, Phosphorylated Insulin Receptor at tyrosine residues 1162 and 1163
JAK2: Tyrosine protein kinase Janus kinase 2
LDL-C: low-density lipoprotein
Nrf2: nuclear factor [erythroid-derived 2]-like 2
PARP-1: Poly [ADP-ribose] polymerase 1
PBS: phosphate buffered saline
PD: Parkinson’s Disease
PPAR-γ: peroxisome proliferator-activated receptor gamma
SG: sitagliptin
SOCS3: suppressor of cytokine signaling 3
STAT3[pY705]: *p*-STAT3, phosphorylated signal transducer and activator of transcription 3 at tyrosine residue 705
STZ: streptozotocin
T2DM: type 2 diabetes
TC: total cholesterol
TGs: triglycerides
